# QUAD SHOT radiotherapy for palliation in advanced cancer: current status and future directions

**DOI:** 10.3389/fonc.2026.1776799

**Published:** 2026-04-15

**Authors:** Xia Liu, Zheng-Yun Du, Zhao-Feng Ning, Jian-Liang Liu, Yun-Long Hu, De-Bo Huang

**Affiliations:** Department of Radiation Oncology, Tai’an Cancer Hospital, Tai’an, Shandong, China

**Keywords:** accelerated radiotherapy, hypo-fractionation, palliative treatment, radiotherapy, symptom control

## Abstract

QUAD SHOT radiotherapy (QS-RT) is a periodic, short-course, hypo-fractionated radiotherapy regimen that originated in the 1980s. It is widely used in Europe and the United States, but less so in Asia. Many clinical studies have shown that QS-RT offers significant advantages in improving quality of life and local control and has a favorable safety profile. This article systematically reviews the origin, biological mechanisms, clinical applications, efficacy, and safety of QS-RT based on existing clinical studies, discusses its clinical advantages and limitations, and considers how to optimize future treatment plans.

## Introduction

Patients with advanced cancer generally have poor performance status and are often accompanied by symptoms such as bleeding, dysphagia, shortness of breath, and pain, which make standard radical radiotherapy intolerable. Although hypofractionated short-course palliative radiotherapy (3Gy×10f) may provide temporary symptom relief, the incidence of grade ≥3 toxicity is high (1). Therefore, there is an urgent clinical need for time-efficient treatment approaches that provide rapid symptom relief and have minimal side effects for patients with advanced cancer. QS-RT is a periodic, short-course hypofractionated radiotherapy regimen that originated in the 1980s. This technique typically involves the delivery of 3.5-3.7 Gy twice daily for two consecutive days, for a total dose of 14–14.8 Gy, at intervals of 3–4 weeks. It can be repeated for three or more cycles. This article reviews relevant clinical studies on QS-RT.

## Origin and biological mechanism of QUAD SHOT radiotherapy

In the 1970s, the M.D. Anderson Cancer Center first reported a short-course hypofractionated radiotherapy regimen for advanced pelvic cancers, in which 10 Gy was delivered in a single fraction for three cycles at 4-week intervals. Although the palliative effect was significant (2), the incidence of grade ≥3 late gastrointestinal toxicity reached 49% (3). Subsequently, the phase II RTOG 8502 clinical study (4) modified the regimen to a periodic hypofractionated scheme, namely the QS-RT regimen, which delivered 3.7 Gy per fraction twice daily at intervals of more than 6 h, for a total dose of 14.8 Gy over two consecutive days, repeated after 3 weeks for a total of three cycles. The results showed that, compared with previous short-course palliative hypofractionated regimens, the tumor response rate was similar, whereas the incidence of grade ≥3 late complications was only 5%. The subsequent phase III RTOG 8502 clinical study (5) conducted a randomized controlled trial comparing 2-week and 4-week intervals and showed no significant differences in tumor response rates or late complications between the two intervals, although acute toxicity increased with the 2-week interval. Long-term follow-up indicated that the incidence of grade ≥3 late complications with QS-RT was 6% (6). In 2005, Corry and colleagues (7) conducted a prospective study of QS-RT for advanced head and neck cancers and, for the first time, introduced this treatment technique in the literature under the name “QUAD SHOT.

The single-fraction dose of radiotherapy is closely associated with late toxicity. Because the single-fraction dose of QS-RT (3.5–3.7 Gy) is relatively low, late toxicity is reduced. After the first cycle of QS-RT, oxygen-sensitive cells are eliminated, improving blood supply to the remaining hypoxic tumor tissue and thereby increasing the sensitivity of the remaining tumor tissue to subsequent cycles of radiotherapy. During the interval, residual tumor cells undergo redistribution in the cell cycle and become more sensitive to radiotherapy. The proliferation rate of mucosal epithelial cells in the oral cavity and pharynx is very rapid, and the biologically effective dose (BED) for each cycle is below the threshold for inducing mucositis. The radiotherapy interval provides sufficient time for mucosal stem cells to repopulate and repair damaged cells, thereby reducing the risk of severe radiation-induced mucositis. Theoretically, tumors may also proliferate during the interval, but the time required for cancer cell proliferation is longer, and there is no significant difference in tumor response rates between the 2-week and 4-week intervals (5, 6). Based on a tumor tissue α/β ratio of 10, the total BED for completion of all three cycles of QS-RT is 60.8 Gy, with an equivalent dose in 2 Gy fractions (EQD2) of 50.7 Gy (8). Shinde et al. (9) reported a case of advanced oropharyngeal squamous cell carcinoma that progressed after four cycles of ipilimumab combined with nivolumab. After QS-RT was applied to the primary site and cervical lymph nodes, the untreated lung metastases also regressed significantly, indicating that QS-RT may induce an abscopal effect.

## Implementation of QUAD SHOT radiotherapy

In the two-dimensional era, target volume definition was based mainly on physical examination, and the target volume for QS-RT was often imprecise, typically extending 1–2 cm beyond the gross tumor. In the three-dimensional era, the gross tumor volume (GTV) is delineated accurately based on imaging, with a clinical target volume (CTV) generated by expanding the GTV by 3–5 mm and a planning target volume (PTV) generated by expanding the CTV by 2–3 mm (10), or by directly expanding the GTV by 0.5–1.0 cm (11). Because QS-RT is primarily used for palliative treatment of advanced tumors, the target volume focuses mainly on the field involved without elective irradiation. For patients with skin invasion or ulceration, tissue compensators should be added to increase the skin dose and improve local control. Radiotherapy techniques may include three-dimensional conformal radiotherapy (3D-CRT), intensity-modulated radiotherapy (IMRT), volumetric modulated arc therapy (VMAT), or proton therapy. Each treatment cycle requires repeat simulation, target volume delineation, and treatment planning. Online adaptive radiotherapy may also be performed. The dose limit for organs at risk (OARs), such as the spinal cord, brainstem, and optic chiasm, should not exceed 13 Gy per cycle of QS-RT, whereas for other OARs, the “as low as reasonably achievable” (ALARA) principle should be followed (12). For patients with a history of radiotherapy, the maximum dose limit for the spinal cord and brainstem is 12 Gy over three cycles or a cumulative maximum dose of 50Gy (13).

## Clinical applications

### Head and neck cancers

Advanced head and neck cancers often present with pain, dysphagia, hoarseness, shortness of breath, stridor, nasal obstruction, neck masses, bleeding, and trismus. QS-RT is widely used in the palliative treatment of advanced head and neck cancers, with minimal toxicity and efficacy similar to that of other palliative treatments (14). Piras et al. (8) conducted a feasibility analysis of hypofractionated radiotherapy for advanced head and neck cancers and suggested that QS-RT can serve as an important palliative option. However, among patients who previously received radiotherapy for head and neck cancer, the proportion who complete all three cycles of QS-RT is relatively low, and progression-free survival (PFS) is shorter, with greater toxicity; therefore, careful patient selection is warranted (1, 15). Recent clinical studies on QS-RT for advanced head and neck cancers are summarized in **Table 1**.

**Table 1:** Recent clinical studies related to QS-RT for advanced head and neck cancers.

Author and year	Number of cases	Combined regimen	Response rate (%)	≥ Grade 3 toxicity (%)	Survival (months)
Fan et al., 2020 (16)	166	50% concurrent chemotherapy, targeted or immunotherapy	66	10.8	6.4
Toya et al., 2020 (17)	34	None	85	0	5.7
Upadhyay et al. 2024 (18)	70	57% concurrent immunotherapy 27.1% concurrent chemotherapy or targeted	71.4	23	9-10
Saha et al., 2024 (19)	116	None	77	0	12
Toya et al. 2024 (10)	105	12% concurrent immunotherapy, targeted or chemotherapy	88	2	6.8
Liu et al., 2025 (20)	10	None	80	10	3.75
Nikolaidou et al. (1)	51	25% with concurrent chemotherapy, immunotherapy or targeted	41	4	5.9

### Pelvic cancers

Advanced pelvic malignancies arise mainly from gynecological, gastrointestinal, and urological cancers and often present with abdominal distension, pain, bleeding, intestinal obstruction, urinary obstruction, and lower extremity edema. The RTOG 8502 study (5) showed that shortening the QS-RT interval from 4 to 2 weeks did not improve tumor response rates. The overall response rate was 42% among patients who completed all 3 cycles, and the 1-year survival rate was 40% in the 2-week interval group among patients with a KPS ≥80. Grade ≥3 acute toxicity was 7% in the 2-week interval group and 0% in the 4-week interval group, and the incidence of grade ≥3 late toxicity was approximately 6%. Kiattikul et al. (21) reported that among 36 patients with advanced cervical cancer treated with QS-RT combined with brachytherapy, the local control rate reached 75%, the pelvic control rate reached 60%, and the median overall survival was 18.6 months; 16% of patients experienced grade 3–4 gastrointestinal toxicity. Yamamoto et al. (22) reported a case of penile metastasis from colon cancer causing pain, urinary retention, and persistent penile erection, in which the patient experienced pain relief and tumor shrinkage after QS-RT and regained the ability to urinate independently until death.

### Other cancers

Patients with advanced breast cancer often present with ulcerative tumors of the breast or chest wall due to skin breakdown, leading to persistent pain, bleeding, foul-smelling discharge, and infection, which severely affect quality of life and result in low self-esteem, depression, and perceived social isolation. Kil et al. (23) reported that among 17 patients with advanced breast cancer who received QS-RT in addition to systemic treatment, 100% experienced significant relief of pain, bleeding, and exudation within 1 week after the first cycle of radiotherapy. After completion of the third cycle, 82% achieved complete symptom relief, and 12% achieved near-complete relief. The 1-year tumor control rate within the target volume was 88%, with 53% achieving complete response (CR) and 35% achieving partial response (PR), and no grade ≥3 toxicity was observed during or after treatment. At a median follow-up of 12 months, 9 patients were still alive. Recurrent metastatic sarcomas are highly heterogeneous and lack effective treatment options, and local radiotherapy is often used for local control. Lee et al. (24) reported a subjective overall response rate of 70% among 28 patients with recurrent metastatic sarcoma treated with proton QS-RT, with a median progression-free survival of 11 months. Univariate analysis showed that receiving four cycles and continuing systemic treatment after radiotherapy were associated with improved local progression-free survival, and 3 patients experienced grade ≥3 toxicity. Advanced cutaneous squamous cell carcinoma can invade adjacent organs, leading to disfigurement and dysfunction. Graczyk et al. (25) reported that among 9 patients with unresectable advanced non-melanoma skin cancer, QS-RT provided excellent local control and significant relief of bleeding, pain, and exudation, with no grade ≥3 toxic reactions. Kil et al. (12) reported a case of unresectable advanced facial cutaneous squamous cell carcinoma causing disfigurement, in which excellent local control was achieved after QS-RT.

In summary, QS-RT was initially applied to advanced pelvic malignancies but is most widely used in head and neck cancers. Analysis of the above studies indicates that QS-RT significantly improves patient compliance and reduces the economic burden on the health care system. In addition, QS-RT has mild side effects, and the patient’s quality of life improves significantly. The symptom relief rate is comparable to that of traditional palliative regimens. However, it is associated with fewer side effects, providing a safer option for the palliative treatment of advanced head and neck cancers. Based on the data from the above studies, version 1 of the 2026 National Comprehensive Cancer Network (NCCN) Guidelines for Head and Neck Cancers also recommends the QS-RT regimen as a palliative radiotherapy option for patients with head and neck cancer (26).

## Combined treatment regimens

### Combined chemotherapy or targeted therapy

Carrascosa et al. (27) reported that among 19 patients with head and neck or pelvic cancers, 5 achieved complete response (CR) and 13 achieved partial response (PR) after the addition of paclitaxel to QS-RT. The symptom relief rate was 94.7%, and 57.9% of patients achieved complete symptom control. Gamez et al. (28) reported that among 21 patients with locally advanced head and neck malignancies and significant symptoms, those treated with QS-RT combined with carboplatin or cetuximab achieved an objective response in 19 cases, including 5 CRs. All 16 patients who completed all three cycles achieved symptom relief, and most experienced complete symptom control after treatment.

### Combined immunotherapy

Higgins et al. (29) reported that among three patients with advanced anal mucosal malignant melanoma treated with QS-RT combined with ipilimumab and nivolumab, all achieved durable local control. Upadhyay et al. (18) showed that local control of head and neck cancers was significantly improved with QS-RT combined with immunotherapy, but no significant differences were observed in distant control or survival. Brockwell et al. (13) reported a case of extensive facial skin cancer in which the patient achieved CR of the primary lesion after treatment with cemiplimab combined with QS-RT, with no evidence of recurrence 1 year after reconstructive surgery. Okada et al. (30) reported a patient with skin metastasis from parotid gland cancer who experienced significant symptom relief after QS-RT combined with chemotherapy and immunotherapy, with no serious adverse events. Therefore, Hughes et al. (31) initiated a clinical study to clarify the overall response rate of pembrolizumab combined with QS-RT and to explore the mechanisms by which QS-RT enhances immune effects.

### Preoperative treatment

Nguyen et al. (32) reported that among 12 patients who underwent surgery after QS-RT, 1 achieved CR and 4 achieved PR. Leming et al. (33) reported a male patient with tongue cancer who underwent only one cycle of QS-RT before surgery, with pathology showing CR. Takahashi et al. (34) reported a female patient with unresectable giant parotid gland cancer who underwent successful surgical resection after marked tumor shrinkage following two cycles of QS-RT combined with pembrolizumab.

In summary, compared with radiotherapy alone, QS-RT combined with other treatment regimens, especially immune checkpoint inhibitors, shows improved local response rates and a trend toward prolonged survival. However, the above studies are mostly small retrospective studies or case reports, and larger randomized controlled trials are needed.

## Clinical advantages and efficacy influencing factors of QUAD SHOT radiotherapy

### Clinical advantages

QS-RT is used primarily in patients with poor performance status, those who refuse other treatments, those with bulky tumors, those who are unsuitable for high-intensity definitive radiotherapy, and those with a short life expectancy. The main treatment goals are symptom control and improvement of quality of life rather than prolongation of survival. The overall treatment duration is short and does not delay systemic treatment. Patients experience rapid symptom relief and better tolerance (12). Because the total number of treatment sessions is lower, medical costs are reduced, and medical resources are conserved. As the tumor shrinks, the target volume for subsequent cycles can be reduced further, thereby lowering the dose to OARs and resulting in milder side effects (35). The number of QS-RT cycles can be adjusted flexibly according to patient performance and tumor response, allowing timely discontinuation of subsequent QS-RT in patients with comorbidity, tumor progression, or rapid deterioration in performance status to avoid ineffective treatment or unnecessary side effects. Patients with a history of radiotherapy can also undergo re-challenge with QS-RT (15, 16).

### Efficacy influencing factors

Although the BED of QS-RT is slightly lower than that of traditional radical radiotherapy regimens, some studies have shown that the complete symptom relief rate among patients who complete the entire QS-RT course is 100%, whereas the complete relief rate among those who receive fewer than 3 cycles is only 44%-50% (36). The Karnofsky performance score is a significant predictor of the number of completed cycles, and in multivariate survival analysis, performance status and histological type are significant variables (5). The use of systemic treatment before and after QS-RT, or proton therapy, may improve prognosis (15, 16). In addition, age, prior radiotherapy history, primary tumor site, T stage, and pathological type are also important factors influencing prognosis (1).

## Limitations of QUAD SHOT radiotherapy

Although combined treatment strategies incorporating QS-RT have been associated with prolonged survival, QS-RT alone does not confer a clear survival advantage over other palliative radiotherapy modalities (14).

QS-RT generally requires at least 3 cycles, necessitating repeated simulations, target volume delineation, and treatment planning, thereby increasing the workload. Furthermore, although the literature on QS-RT includes randomized controlled trials, many QS-RT studies are small, retrospective, or case reports, raising concerns about potential publication bias. There is also a lack of basic and preclinical research, especially regarding changes in the tumor immune microenvironment after QS-RT. For radiosensitive tumors, care must be taken to prevent tumor lysis syndrome (37), and treatment of hollow-organ lesions or tumors invading major blood vessels may be limited because of the risks of perforation and fatal bleeding. Tanaka et al. (38) reported that although tumor-related pain was alleviated after QS-RT, QS-RT-related oral mucositis increased pain, thus emphasizing the need for enhanced oral care during treatment of advanced head and neck cancers.

## Future directions

As a form of pulse radiotherapy, QS-RT is currently applied mainly in the palliative setting, but research on personalized ultrahypofractionated stereotactic adaptive radiotherapy, which is also a form of pulse radiotherapy, has shown considerable promise (39), not only in alleviating tumors but also in generating immune memory and enhancing the efficacy of immunotherapy (40). Future studies could evaluate QS-RT in combination with targeted therapy, immune checkpoint inhibitors, and antibody-drug conjugates to further improve efficacy, aiming to move beyond symptom control toward prolongation of survival.

Additionally, optimizing workflows through rapid online adaptive radiotherapy can reduce workload (20). Increasing the number of QS-RT cycles while ensuring OAR safety can enhance BED. Active exploration of new indications will allow more patients with advanced cancer to benefit.

## Conclusion

Numerous clinical studies have demonstrated that QS-RT provides significant palliative benefits for patients with advanced cancer, including rapid symptom relief, excellent tolerance, a low incidence of side effects, and reduced medical costs. It has promising clinical applications for patients who cannot tolerate definitive radiotherapy and is expected to become an important modality in the palliative setting, benefiting more patients with advanced cancer. Future efforts should focus on conducting large-scale randomized controlled trials to validate and establish this therapeutic approach, as well as on investigations into its underlying mechanisms, expansion into new indications, and optimization of combined treatment modalities.

## References

[1] Nikolaidou E Bostel T Mayer A Wollschläger D Schmidberger H Kaufmann J . Quad shot—a tertiary hospital experience with a palliative radiotherapy regimen for head and neck cancer. Head Neck. (2025) 47:2827–34. doi: 10.1002/hed.28202. PMID: 40457636 PMC12434552

[2] Boulware RJ Caderao JB Delclos L Wharton JT Peters LJ . Whole pelvis megavoltage irradiation with single doses of 1000 rad to palliate advanced gynecologic cancers. Int J Radiat Oncol Biol Phys. (1979) 5:333–8. doi: 10.1016/0360-3016(79)91212-4. PMID: 88436

[3] Spanos JWJ Wasserman T Meoz R Sala J Kong J Stetz J . Palliation of advanced pelvic Malignant disease with large fraction pelvic radiation and misonidazole: final report of RTOG phase I/II study. Int J Radiat Oncol Biol Phys. (1987) 13:1479. doi: 10.1016/0360-3016(87)90314-2. PMID: 2442127

[4] Spanos JW Guse C Perez C Grigsby P Doggett RL Poulter C . Phase II study of multiple daily fractionations in the palliation of advanced pelvic Malignancies: preliminary report of RTOG 8502. Int J Radiat Oncol Biol Phys. (1989) 17:659. doi: 10.1016/0360-3016(89)90120-x. PMID: 2476426

[5] Spanos WJJ Perez CA Marcus S Poulter CA Doggett RL Steinfeld AD . Effect of rest interval on tumor and normal tissue response--a report of phase III study of accelerated split course palliative radiation for advanced pelvic Malignancies (RTOG-8502). Int J Radiat Oncol Biol Phys. (1993) 25:399–403. doi: 10.1016/0360-3016(93)90059-5. PMID: 7679668

[6] Spanos WJJ Clery M Perez CA Grigsby PW Doggett RL Poulter CA . Late effect of multiple daily fraction palliation schedule for advanced pelvic Malignancies (RTOG 8502). Int J Radiat Oncol Biol Phys. (1994) 29:961–7. doi: 10.1016/0360-3016(94)90389-1. PMID: 8083097

[7] Corry J Peters LJ Costa ID Milner AD Fawns H Rischin D . The ‘QUAD SHOT’—a phase II study of palliative radiotherapy for incurable head and neck cancer. Radiother Oncol. (2005) 77:137–42. doi: 10.1016/j.radonc.2005.10.008. PMID: 16260054

[8] Piras A Boldrini L Menna S Venuti V Pernice G Franzese C . Hypofractionated radiotherapy in head and neck cancer elderly patients: a feasibility and safety systematic review for the clinician. Front Oncol. (2021) 11:761393. doi: 10.3389/fonc.2021.761393. PMID: 34868976 PMC8633531

[9] Shinde A Novak J Freeman ML Glaser S Amini A . Induction of the abscopal effect with immunotherapy and palliative radiation in metastatic head and neck squamous cell carcinoma: a case report and review of the literature. Cureus. (2019) 11:e4201. doi: 10.7759/cureus.4201. PMID: 31114720 PMC6505722

[10] Toya R Fukugawa Y Saito T Matsuyama T Yoshida R Murakami D . Radiation therapy oncology group 8502 “QUAD shot” regimen using volumetric modulated arc therapy for incurable head and neck cancer. Oral Oncol. (2024) 151:106752. doi: 10.1016/j.oraloncology.2024.106752. PMID: 38518555

[11] Kil WJ . Rapid and durable symptom palliation with quad shot radiation therapy to nonosseous metastatic/recurrent cancer in elderly or frail patients in a rural community clinic. Adv Radiat Oncol. (2022) 7:100871. doi: 10.1016/j.adro.2021.100871. PMID: 35079665 PMC8777150

[12] Kil WJ Camphausen K Cho IH . Clinical and radiobiological consideration of cyclical hypofractionated radiation therapy also known as QUAD shot for neglected skin cancer disfiguring the face of a non-compliant patient who was refusing surgery and protracted radiation therapy: case report. Radiat Oncol J. (2019) 37:143–8. doi: 10.3857/roj.2019.00248. PMID: 31266294 PMC6610004

[13] Brockwell M Husain M Verschraegen C Wu R Tinoco G . Case report: the power of immunotherapy in advanced cutaneous squamous cell carcinoma. Front Oncol. (2023) 12:1081118. doi: 10.3389/fonc.2022.1081118. PMID: 36686737 PMC9846513

[14] Chen AM Vaughan A Narayan S Vijayakumar S . Palliative radiation therapy for head and neck cancer: toward an optimal fractionation scheme. Head Neck. (2008) 30:1586–91. doi: 10.1002/hed.20894. PMID: 18798313

[15] Lee A Woods R Mahfouz A Kitpanit S Cartano O Mohamed N . Evaluation of proton therapy reirradiation for patients with recurrent head and neck squamous cell carcinoma. JAMA Netw Open. (2023) 6:e2250607. doi: 10.1001/jamanetworkopen.2022.50607. PMID: 36689229 PMC9871797

[16] Fan D Kang JJ Fan M Wang H Lee A Yu Y . Last-line local treatment with the quad shot regimen for previously irradiated head and neck cancers. Oral Oncol. (2020) 104:104641. doi: 10.1016/j.oraloncology.2020.104641. PMID: 32182548 PMC8480112

[17] Toya R Saito T Matsuyama T Watakabe T Yamaguchi K Murakami D . QUAD shot: an effective cyclical hypofractionated palliative radiotherapy for salivary gland carcinoma. BJR Case Rep. (2020) 6:20190132. doi: 10.1259/bjrcr.20190132. PMID: 33299577 PMC7709058

[18] Upadhyay R Gogineni E Tocaj G Ma SJ Bonomi M Bhateja P . Palliative quad shot radiation therapy with or without concurrent immune checkpoint inhibition for head and neck cancer. Cancers (Basel). (2024) 16:1049. doi: 10.3390/cancers16051049. PMID: 38473406 PMC10931206

[19] Saha S Mallick I Mukherjee P Chakraborty S Bhattacharyya T S MA . Are oral cancers effectively palliated with radiotherapy? Outcomes of treatment with a modifiedQUAD SHOT regimen. Head Neck. (2024) 46:1270–9. doi: 10.1002/hed.27730. PMID: 38528774

[20] Liu W Schiff JP Hassanzadeh C Miller K Hatscher C Beckert R . Palliative expeditiously adaptive quad shot radiotherapy for head and neck cancers (PEAQ-RT). Clin Transl Radiat Oncol. (2025) 54:101012. doi: 10.1016/j.ctro.2025.101012. PMID: 40703666 PMC12284596

[21] Kiattikul CL Narayan K Bernshaw D Dyk SV Tzovaras A Lin MY . Tumor control after palliative hypofractionated, “quad-shot,” external beam radiotherapy followed by brachytherapy: an effective approach in medically compromised and/or elderly patients with cervix cancer. J Cancer Res Ther. (2022) 18:173–9. doi: 10.4103/jcrt.JCRT_1346_20. PMID: 35381780

[22] Yamamoto A Ieki H Shimamura M Tsujiura M Yokoe T Sanuki N . Symptom palliation with QUAD shot radiation therapy to penile metastasis derived from descending colon cancer: a case report. Int Cancer Conf J. (2023) 12:210–5. doi: 10.1007/s13691-023-00604-y. PMID: 37251007 PMC10212838

[23] Kil WJ Smith W Muchnik E Collins R Cousins D . Adaptive repeat quad shot radiation therapy for uncontrolled symptomatic fungating or skin-infiltrating primary and regional nodes in patients with metastatic breast cancer: durable in-field tumor control without interrupting systemic therapy. Adv Radiat Oncol. (2025) 10:101842. doi: 10.1016/j.adro.2025.101842. PMID: 40761685 PMC12319245

[24] Lee A Kang JJ Bernstein H Marqueen KE Neal B Kelly CM . Proton radiotherapy for recurrent or metastatic sarcoma with palliative quad shot. Cancer Med. (2021) 10:4221–7. doi: 10.1002/cam4.3646. PMID: 34085781 PMC8267151

[25] Graczyk Ł Bibik R Woźniak A Jesień-Lewandowicz E Ciupis J Szyburski M . QUAD SHOT – an effective hypofractionated palliative radiotherapy in patients with non-melanoma skin cancer. A single-institution experience. Contemp Oncol (Pozn). (2023) 27:80–9. doi: 10.5114/wo.2023.129462. PMID: 37794986 PMC10546968

[26] National Comprehensive Cancer Network . NCCN Clinical Practice Guidelines in Oncology: Head and Neck Cancers, Version 1.2026. Plymouth Meeting, PA: National Comprehensive Cancer Network; (2026).

[27] Carrascosa LA Yashar CM Paris KJ Larocca RV Faught SR Spanos WJ . Palliation of pelvic and head and neck cancer with paclitaxel and a novel radiotherapy regimen. J Palliat Med. (2007) 10:877. doi: 10.1089/jpm.2006.0192. PMID: 17803408

[28] Gamez ME Agarwal M Hu KS Lukens JN Harrison LB . Hypofractionated palliative radiotherapy with concurrent radiosensitizing chemotherapy for advanced head and neck cancer using the “QUAD-SHOT regimen. Anticancer Res. (2017) 37:685–92. doi: 10.21873/anticanres.11364. PMID: 28179317

[29] Higgins MJ Alipour R Pope K Ung KA Kok DL Chua MS . QUAD SHOT radiotherapy and doublet immunotherapy in the management of anal mucosal melanoma: a case series of efficacy and toxicity of a novel treatment approach and a review of the literature. Clin Colorectal Cancer. (2022) 21:e179–86. doi: 10.1016/j.clcc.2022.03.001. PMID: 35396149

[30] Okada K Takahashi S Endo M Fukuda Y Ogawa K Kawahara M . The efficacy of radiation therapy using the quad shot regimen in cutaneous metastasis from parotid gland cancer: a case report. Clin Case Rep. (2023) 11:e7687. doi: 10.1002/ccr3.7687. PMID: 37469363 PMC10352556

[31] Hughes RT Gebeyehu RR Kalada JM Lycan TW Frizzell BA Kinney RD . Quad-shot-immunotherapy: quad-shot radiotherapy with pembrolizumab for advanced/recurrent head and neck cancer. Future Oncol. (2023) 19:1523–34. doi: 10.2217/fon-2022-1146. PMID: 37199326 PMC10463211

[32] Nguyen M Hsieh M Henson C Krempl G . Neoadjuvant QUAD shot for downstaging or temporizing locally advanced oral cavity cancer prior to definitive surgery. Oral Oncol. (2022) 133:106029. doi: 10.1016/j.oraloncology.2022.106029. PMID: 35870330 PMC12927544

[33] Leming AB Johnston AL Krempl GA Fowle EJ Morton DJ Henson CE . Pathologic complete response following low-dose radiation for advanced oral cavity cancer in a patient with human immunodeficiency virus. J Otolaryngol Head Neck Surg. (2022) 51:37. doi: 10.1186/s40463-022-00586-6. PMID: 36192808 PMC9531370

[34] Takahashi S Endo M Nagatomo T Onaga R Yamaguchi H Yamamoto R . Successful preoperative QUAD SHOT for bulky parotid carcinoma: potential preoperative ultra-hypofractionated radiotherapy for conversion surgery. Case Rep Oncol. (2023) 16:218–26. doi: 10.1159/000529829. PMID: 37069898 PMC10105323

[35] Choudhary A Gupta A . Conventional fractionation versus quad shot in advanced head-and-neck cancers: a randomized controlled trial. Indian J Palliat Care. (2019) 25:527. doi: 10.4103/IJPC.IJPC_209_18. PMID: 31673207 PMC6812420

[36] Paris KJ Spanos WJJ Lindberg RD Jose B Albrink F . Phase I-II study of multiple daily fractions for palliation of advanced head and neck Malignancies. Int J Radiat Oncol Biol Phys. (1993) 25:657–60. doi: 10.1016/0360-3016(93)90012-k. PMID: 7681051

[37] Newbury P Ganguly R Henson C . Case report: tumor lysis syndrome after quad shot in diffuse large b-cell lymphoma. Adv Radiat Oncol. (2023) 8:101265. doi: 10.1016/j.adro.2023.101265. PMID: 37332529 PMC10275961

[38] Tanaka O Naganawa K Matsuzuka T Ehara Y Hasegawa Y Kiryu T . Impact of stomatitis on pain relief and nutrition in palliative radiotherapy using quad shot: a prospective study. J Radiat Res. (2025) 66:666–72. doi: 10.1093/jrr/rraf058. PMID: 40988098 PMC12648052

[39] Moore C Hsu C Chen W Chen BPC Han C Story M . Personalized ultrafractionated stereotactic adaptive radiotherapy (PULSAR) in preclinical models enhances single-agent immune checkpoint blockade. Int J Radiat Oncol Biol Phys. (2021) 110:1306–16. doi: 10.1016/j.ijrobp.2021.03.047. PMID: 33794306 PMC8286324

[40] Sezen D Barsoumian HB He K Hu Y Wang Q Abana CO . Pulsed radiotherapy to mitigate high tumor burden and generate immune memory. Front Immunol. (2022) 13:984318. doi: 10.3389/fimmu.2022.984318. PMID: 36275767 PMC9582356

